# MXene, protein, and KCl-assisted ionic conductive hydrogels with excellent anti-freezing capabilities, self-adhesive, ultra-stretchability, and remarkable mechanical properties for a high-performance wearable flexible sensor†

**DOI:** 10.1039/d4ra02707h

**Published:** 2024-07-09

**Authors:** Irfan Ijaz, Aysha Bukhari, Ezaz Gilani, Ammara Nazir, Hina Zain, Attia Shaheen, Mohammed Rafi Shaik, Mohamed E. Assal, Mujeeb Khan

**Affiliations:** a School of Chemistry, Faculty of Basic Sciences and Mathematics, Minhaj University Lahore Lahore 54700 Pakistan iffichemixt266@gmail.com ayshabukhari.che@mul.edu.pk; b Department of Chemistry, University of Cincinnati OH 45221 USA; c Institute for Advanced Study, Shenzhen University Shenzhen Guangdong P.R. China; d Department of Chemistry, College of Science, King Saud University P. O. Box 2455 Riyadh 11451 Saudi Arabia

## Abstract

Developing a hydrogel with switchable features and freeze tolerance is remarkably significant for designing flexible electronics to adjust various application needs. Herein, MXenes, AFPs (antifreeze proteins), and potassium chloride (KCl) were introduced to a polyacrylamide (PAM) polymer network to design an anti-freezing hydrogel. The ionic hydrogels are characterized by excellent ionic conductivity, presenting adjustable properties of remarkable mechanical strength and self-adhesion to meet individualized application demands. The capability of KCl and AFPs to inhibit ice crystals gives hydrogels with anti-icing properties under a low-temperature atmosphere. The PAM/MXene_15_/AFP_30_/KCl_15_ hydrogel demonstrated negligible hysteresis behavior, quick electromechanical response (0.10 s), and excellent sensitivity (gauge factor (GF) = 13.1 within the strain range of 1200–2000%). The resulting hydrogel could be immobilized on the animal or human skin to detect different body movements and physiological motions, offering reproducibility and precise accuracy as primary advantages. The approach of developing materials with tunable features, along with inorganic salt and the fish-inspired freeze-tolerance method, offers a new prospect for wearable gadgets.

## Abstract 2

The PAM/MXene_15_/AFP_30_/KCl_15_ hydrogel demonstrated negligible hysteresis behavior, quick electromechanical response (0.10 s), and excellent sensitivity (gauge factor (GF) = 13.1 within the strain range of 1200–2000%).

## Graphical abstract

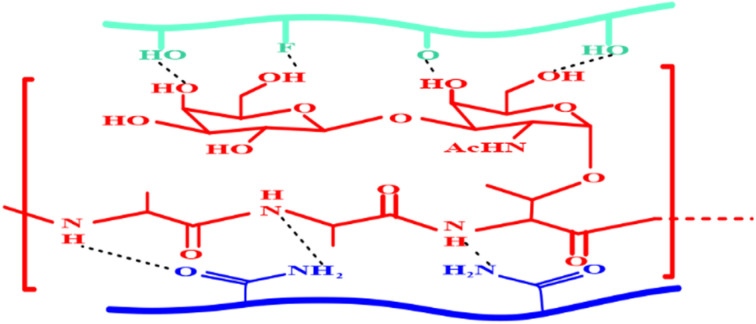

## 1 Introduction

High sensitivity with excellent flexibility has been the quest for sensors to emulate the human skin's function and use in motion detection,^1–3^ intelligent robotics,^4,5^ real-time kinematics investigation,^6,7^ and personal healthcare monitoring.^8^ Conductive hydrogels are currently believed to be promising for developing flexible electronics because of their excellent characteristics of mechanical compliance,^9^ super stretchability,^10^ and high conductivity.^11^ However, most hydrogel-based sensors face issues related to non-conductance caused by freezing, consequently significantly hindering their use in low-temperature conditions. Adding low-volatile solvents into the hydrogel is considered an effective approach to increasing its anti-freezing capability.^12^ However, it could sacrifice biocompatibility, conductivity, and mechanical properties.^13^ However, there is a great challenge to design an approach to prepare hydrogels with excellent anti-freezing properties without compromising its original properties.

In our nature, different types of fish have evolved to prevent freezing for survival in the sea at low-temperature conditions, anticipated to their antifreeze proteins (AFPs) in the fish's body.^14^ According to previous reports, antifreeze proteins have distinctive capabilities in modulating the ice crystallization phenomena by selectively creating bonds with the surfaces of ice.^15,16^ Motivated by these results, we anticipated that incorporating AFPs in a well-developed network would significantly improve the mechanical properties and impart the anti-icing and self-adhesive capabilities to hydrogels.

Conductive hydrogels are commonly categorized into ionic conductive and electronic conductive hydrogels. Ionic conductive hydrogel is a type of conductive hydrogel which are conducted through the directional movement of free ions and exhibits a broader sensing range compared to electronic conductive hydrogels.^17^ Furthermore, signals frequently transferred by ions in biological systems and ionic conductive hydrogels exhibit significant potential for utilization in implantable or wearable devices because of their high degree of free ions.^18^ Ionic conductive hydrogels can be prepared by employing salts and hydroxyl-rich polymers.^19^ However, the addition of salt (KCl) provides the hydrogel with outstanding anti-freezing properties and ionic conductivity. Hydroxy species are readily attached to other materials through hydrogen bonding, so hydrogels frequently exhibit tunable mechanical characteristics, and the hydroxy species assist in improving the adhesion of hydrogels. MXene, as a 2D material with –OH groups exhibiting larger specific surface area, superior mechanical properties, and elevated electrical conductivity, is considered the best candidate for the fabrication of ionic conductive hydrogels.^20^ For instance, Ma *et al.* prepared a PAM-sodium alginate-MXene-sucrose (PGMS) hydrogel with outstanding self-adhesion and stretchability, which indicated greater strain sensitivity for differentiating different subtle or strong motions of the human body.^21^ Despite their advantages, the utilization of MXene is significantly restricted by their tendency to oxidation and aggregation of MXene in aqueous conditions.^22^ Such issues are prone to produce inevitable outcomes such as deterioration of sensing ability and decline in mechanical strength. Thus, it remains a challenge to design MXene-based ionic conductive hydrogels with high conductivity and mechanical properties.

The primary goal of this study is to address all the above-mentioned challenges by developing a biocompatible and anti-icing hydrogel with switchable characteristics of mechanical strength and self-adhesion to construct the wearable sensor. PAM/MXene/AFP/KCl ionic hydrogels were synthesized, and hydrogen bonding and strong hydration of KCl greatly enhanced the mechanical strength of the hydrogels, attaining the highest elongation at break of 4891% under strain. AFPs and MXene could effectively interact with polymer chains through hydrogen bonding and significantly improve the self-adhesion and strength of the hydrogels. Furthermore, the existence of salt ions gave the hydrogels sufficient conductivity, promoting their capability to act as sensors for monitoring strain variations with negligible electric hysteresis, rapid response, and greater sensitivity. Notably, the hydrogels indicated exceptional anti-freezing properties achieved through ice crystal inhibition of KCl and AFPs. Consequently, the PAM/MXene/AFP/KCl hydrogel sensors have ability to be affixed to the skin of humans to monitor physiological signals and bodily motions as well as to detect distinct hardness and weight of objects. PAM/MXene/AFP/KCl hydrogel-based sensors were also efficiently utilized to monitor different movements of the mouse, such as tail-wagging, free movement, and larynx vibration.

## 2 Experimental section

### 2.1. Materials

MAX powder (Ti_3_AlC_2_) and antifreeze proteins (AFPs) were purchased from Waltham, MA; USA. Ammonium persulfate (APS), *N*,*N*′-methylenebisacrylamide (MBAA), and potassium chloride (KCl) were obtained from Shanghai Aladdin Co., Ltd.

### 2.2. Preparation of MXene suspension

MXene was synthesized through etching MAX powder (Ti_3_AlC_2_) with lithium fluoride (LiF) and hydrochloric acid (HCl). Particularly, 2 g of LiF was mixed with 20 mL of 10 M HCl solution and stirred for 10 min in a Teflon beaker. Then, 3 g of Ti_3_AlC_2_ MAX powder was gradually introduced into the mixed solution and reacted at 45 °C for 20 h. The mixture was then centrifuged at 3500 rpm for 10 minutes and rinsed multiple times to neutrality with deionized (DI) water. The obtained mixture was centrifuged with deionized water at 4500 rpm for 10 min, and the resulting supernatant was poured off. This phenomenon was repeated until the color of the supernatant turned black. MXene sediments were collected by applying a greater centrifugal speed of 7500 rpm. To prepare the MXene suspension, the previously collected sediments were introduced into 150 mL of deionized water and then sonicated in an Ar environment for 60 min, finally again centrifuged for 60 min at 3500 rpm.

### 2.3. Preparation of PAM/MXene/AFP/KCl hydrogel

**Scheme 1:**
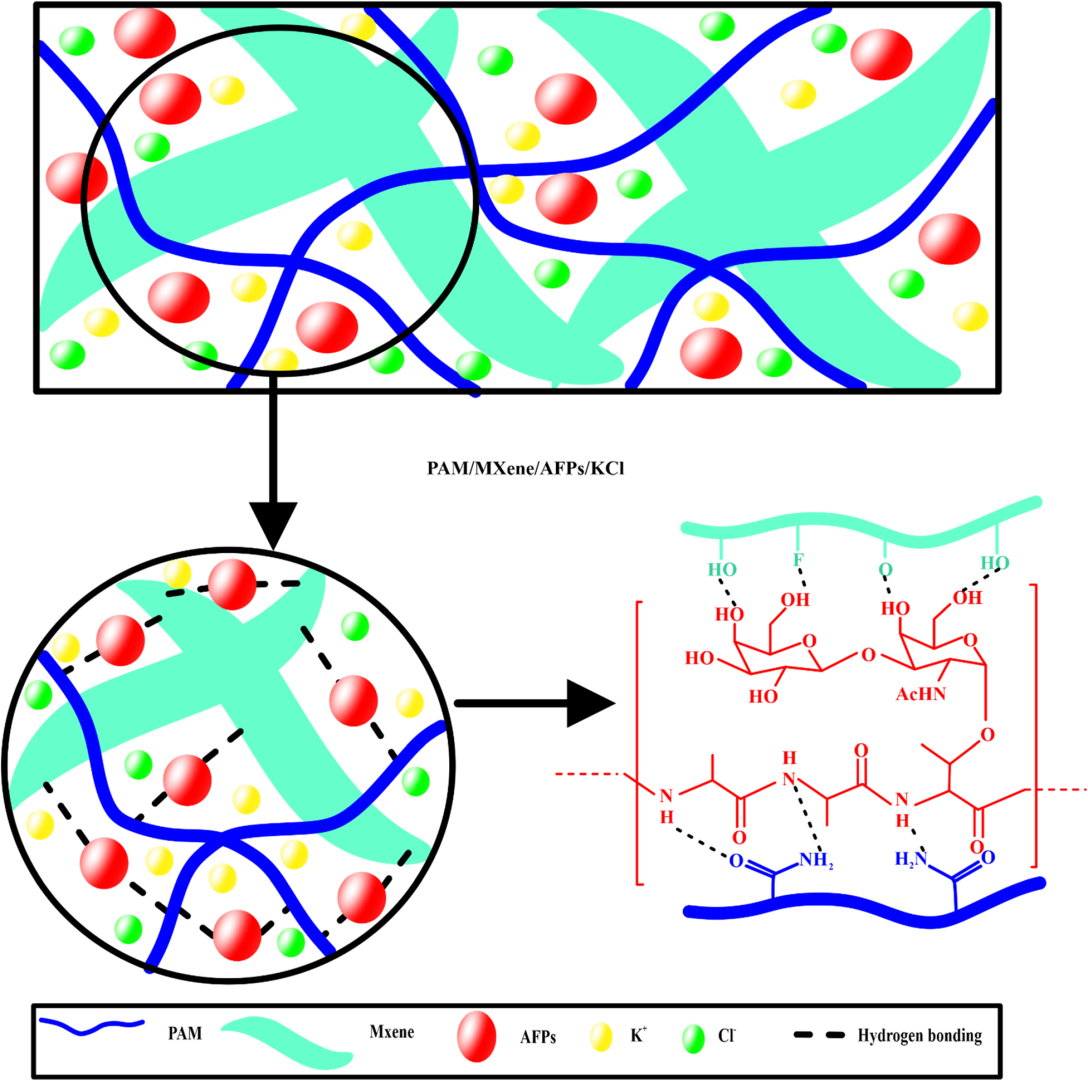
Synthetic mechanism of PAM/MXene/AFPs/KCl hydrogels.

Firstly, the AM monomers were introduced in the MXene suspension to design solution 1. Secondly, AFPs were introduced in the DI water with constant stirring at room temperature to prepare solution 2. Thirdly, solution 3 was prepared by mixing KCl in DI water with constant stirring. Then, solutions 1, 2, and 3 were combined with each other, and MBAA and APS were added to the mixture. Then obtained mixture was introduced into the reaction mold and subjected to copolymerization for 6 h at 50 °C to prepare PAM/MXene_*x*_/AFP_*y*_/KCl_*z*_ hydrogel (Scheme 1). Wherein, *x*, *y*, and *z* were the donated contents of MXene, AFPs, and KCl. For a comparative study, the PAM/MXene, PAM/MXene/AFPs, and PAM/MXene/AFPs/KCl hydrogels were prepared by employing the same strategy with different contents, as illustrated in Table S1.†

### 2.4. Characterization

The chemical structure of the hydrogels was determined utilizing Fourier transform infrared spectroscopy (FTIR; BRUKER TENSOR 27; Germany) at room temperature. The microstructure and surface morphology of hydrogels were studied by using a scanning electron microscope (SEM; JSM-6510, JEOL).

### 2.5. Adhesion test

The detailed process for adhesion test is provided in Text S1.†

### 2.6. Conductivity analysis

The detailed process for conductivity analysis is provided in Text S2.†

### 2.7. Mechanical test

The mechanical strength of PAM/MXene/AFPs/KCl hydrogels was examined by a multifunctional testing machine with a 100 N load sensor (Shimadzu AGS-X, Japan). The PAM/MXene/AFPs/KCl hydrogels were prepared in dumbbell-like shape with thickness of 3 mm and width of 4 mm.

### 2.8. DSC analysis

A differential scanning calorimeter (DSC25, TA, made in the USA) was used to investigate the freeze resistance of PAM/MXene/AFPs/KCl hydrogel at low temperatures. The DSC analysis started at a temperature of 20 °C and then decreased to −60 °C.

### 2.9. Electromechanical measurements

The electrochemical workstation (Vertex C, IVIUM Tech, made in the Netherlands) connected to the tensile tester (MCT-2150, A&D, made in Japan) was employed to investigate the electromechanical response of the hydrogels. The electrochemical workstation recorded output signals as hydrogel was stretched by using a tensile tester. However, the PAM/MXene/AFPs/KCl hydrogels were immobilized on the human body to serve as hydrogel-based sensors to monitor bodily motions by attaching them to metal wires. The equation given below was employed to record the output signal.

1 Δ*R*/*R*_0_ (%) = (*R* − *R*_0_) × 100

where *R*_0_ and *R* denote resistance before and after applying strain on the PAM/MXene/AFPs/KCl hydrogel.

## 3 Result and discussion

### 3.1. FTIR of hydrogels

The FTIR spectra of pristine PAM, PAM/MXene, PAM/MXene/APFs, and PAM/MXene/APFs/KCl hydrogels are illustrated in Fig. S1.† The typical band of PAM hydrogel at 3179 cm^−1^ was related to the NH tensile vibration, and the peaks at 1639 cm^−1^ and 1611 cm^−1^ were primarily attributed to the C=O tensile vibration and the NH bending vibration. With the introduction of MXene, three new peaks at 3425, 1049–1098, and 619 cm^−1^ appeared, which attributed to the OH, C–F, and Ti–O. In addition to the appearance of new peaks in the FTIR spectrum of PAM/MXene, the peaks attributed to the NH and C=O shifted from 3179 cm^−1^ to 3201 cm^−1^, 1611 cm^−1^ to 1619 cm^−1^, and 1639 to 1651 cm^−1^, respectively. These shifts were due to the hydrogen bonds between PAM and MXene. Furthermore, the loading of AFP also led to the transformation of the OH, NH, and C=O, which were primarily due to the formation of hydrogen bonding between AFP and PAM/MXene. The addition of KCl resulted in a transformation of the peak of C=O in the form of –COO^−^ from 1663 cm^−1^ to 1674 cm^−1^, which might be due to the improved hydrogen bonds caused by KCl introduction and the metal complexation or electrostatic interaction between the K^+^ and –COO^−^.

### 3.2. SEM images of hydrogels

**Fig. 1:**
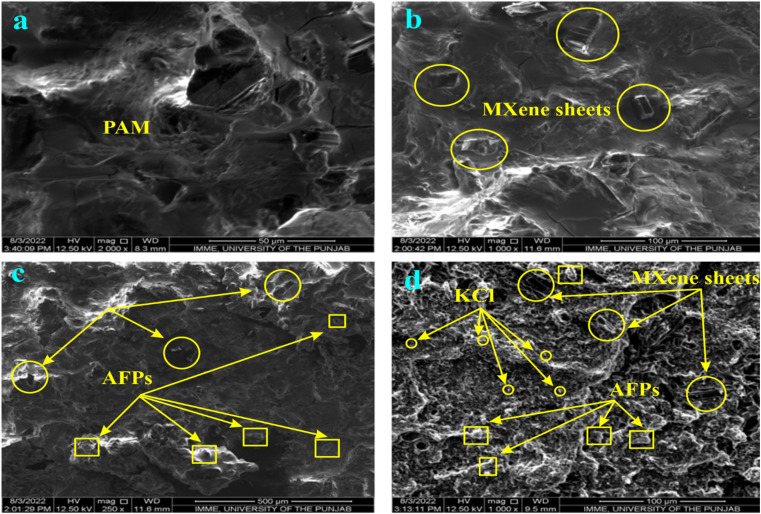
SEM images of pristine PAM (a), PAM/MXene (b), PAM/MXene/APFs (c), and PAM/MXene/APFs/KCl hydrogels (d).

Fig. 1(a–d) exhibits the morphology of different hydrogels. Fig. 1(a) displays that the PAM hydrogel has both smaller and larger pores. Fig. 1(b) exhibited the MXene penetrated in the pores or attached to the surface of PAM through hydrogen bonding between the NH and C=O groups of PAM and OH and F groups of MXene. Fig. 1(c) indicates that MXene sheets and AFP particles were attached to the PAM surface through hydrogen bonding. In addition to MXene and larger AFP particles, smaller KCl particles can be seen in the SEM image of PAM/MXene/AFPs/KCl hydrogel.

### 3.3. Mechanically property

**Fig. 2:**
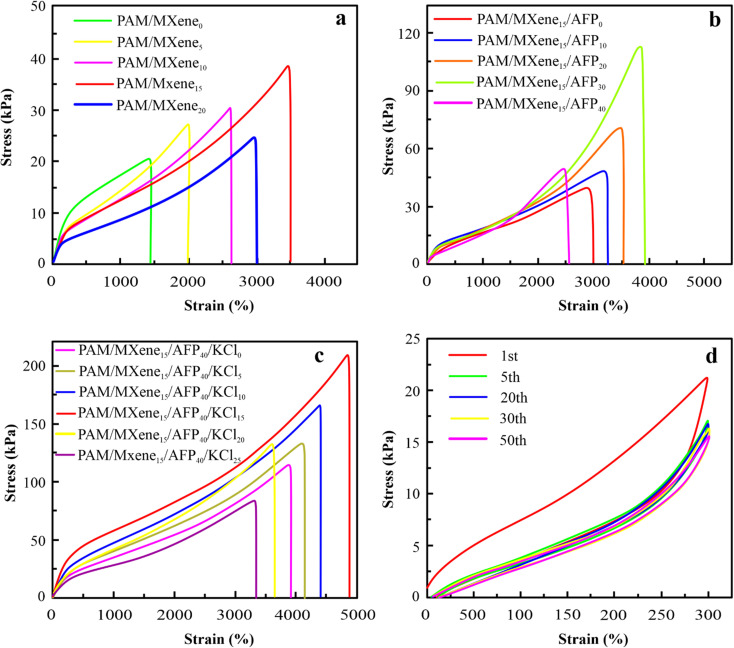
Tensile stress and strain curves of PAM/MXene hydrogels with various MXene contents (a), tensile stress and strain curves of PAM/MXene/AFPs hydrogels with different concentrations of AFPs (b), tensile stress and strain curves of PAM/MXene/AFPs/KCl hydrogels with various KCl contents (c), and cyclic tensile curves of hydrogel for 50 cycles (d).

The mechanical properties of the hydrogel are crucially necessary for their use in hydrogel-based wearable sensors during periods of suffering from huge mechanical loads. The elongation at break of PAM hydrogels improves continually as the content of MXene rises from 0 wt% to 15 wt%, as exhibited in Fig. 2(a). Similarly, the elongation at break of PAM/MXene_15_ hydrogels enhances constantly as the content of AFPs grows from 0 wt% to 30 wt% as displayed in Fig. 2(b). Such increases were because MXene, AFPs, and polymer chains can form hydrogen bonding interactions. As MXene and AFPs content grows, the number of hydrogen bonds developed in hydrogels is enhanced, leading to more polymer chains being connected *via* MXene and AFPs, promoting improved repair of polymer chains suspension as well as reinforcing the hydrogel network. Interestingly, the elongation at break reaches 3500% at 15 wt% MXene content (Fig. S2(a)†). The tensile strength of hydrogel rises from 11.93 to 41.09 kPa, and the toughness enhances from 0.43 to 1.81 MJ m^−3^ with the rise of MXene content (Fig. S2(b and c)†). The drop in Young's modulus of the hydrogel with the rising content of MXene (Fig. S2(d)†) was due to the impact of MXene on the polymerization procedure. Such outcomes were because excessive MXene and AFPs influence the polymerization procedure of the polymer.^23^ According to Fig. 2(b) and S3(a–d),† as the AFPs contents keep rising, tensile strength, the elongation at break, toughness, and Young's modulus of hydrogels slowly increase. Notably, PAM/MXene_15_AFP_30_ hydrogel reached super tensile strength with an elongation at a break of up to 3989%, as exhibited in Fig. S3(a).† Similarly, the tensile strength of MXene_15_AFP_30_ hydrogel increases from 41.09 to 82.39 kPa (Fig. S3(b)†), and the toughness improves from 1.81 to 25.5 MJ m^−3^ with the rise of AFP content (Fig. S3(c)†). Fig. S3(d)† illustrates that Young's modulus of the PAM hydrogel decreased with rising contents of AFPs. Fig. 2(a) shows that as the concentration of MXene increased by 20%, the tensile strength of the PAM/MXene hydrogel decreased. It might be explained by the MXene aggregations at the high concentration.^24^ Similarly, the PAM/MXene_15_/AFP hydrogel's tensile strength declined as the concentration of AFPS increased (Fig. 2(b)). The primary reason for this phenomenon was that AFPS served as a plasticizer in the PAM/MXene_15_/AFP_*X*_ hydrogel network and developed numerous weak bonds with the hydrogel network, including hydrogen bonds.^25,26^

**Fig. 3:**
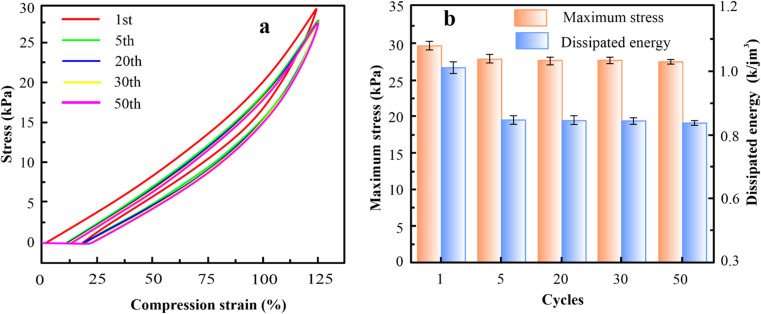
Cyclic compressive curves of PAM/MXene_15_/AFP_30_/KCl_15_ hydrogel for 50 cycles (a), maximum stress and dissipation energy in cyclic compressive curves of PAM/MXene_15_/AFP_30_/KCl_15_ hydrogel for 50 cycles (b).

Thus, the fixed MXene and AFP contents are 15 wt% and 30 wt%, respectively, and the impact of KCl on mechanical strength was investigated *via* tension tests. According to Fig. 2(c) and S4(a–d),† with an increase in KCl contents, the elongation at break, Young's modulus, toughness, and tensile strength of hydrogel increase gradually. This was due to the strong hydration of K generating more non-covalent interactions within the network of hydrogel, such as hydrogen bonding and physical entanglement, which enhanced the mechanical strength of the hydrogels.^27^ Interestingly, PAM/MXene_15_/AFP_30_/KCl_15_ hydrogel reached super tensile strength with an elongation at break of nearly 4891%. Furthermore, with the continuous growth in KCl content, the mechanical characteristics of hydrogel decrease. This occurred due to an excessive amount of KCl binding a higher amount of free water, leading to greater viscosity of the precursor hydrogel solution, and the cage effect at this level was observed due to high viscosity,^28^ which greatly lowers the free radical polymerization efficiency and results in a reduction of mechanical properties. The stability of the mechanical characteristics of PAM/MXene_15_/AFP_30_/KCl_15_ hydrogel was investigated using cyclic compression and cyclic tension analysis. According to Fig. 3(a), the maximum hysteresis was observed in the 1st cycle, and the hysteresis loop was nearly unaltered in the 1st–50th cycles, which suggests that the PAM/MXene_15_/AFP_30_/KCl_15_ hydrogel exhibited a superior energy dissipation capability. Fig. 2(d) and 3(a) exhibited that the maximum tensile and compressive stresses remain near the initial values after 50 consecutive cycles. This implied that the hydrogel's dissipated energy remained high (Fig. 3(b) and S5†). Fig. S6(a and b)† exhibited that the dissipated energy improves slowly with rising strain and compressive strain. This demonstrates that the PAM/MXene_15_/AFP_30_/KCl_15_ hydrogel exhibited quick self-recovery capability and superior fatigue resistance.

### 3.4. Self-adhesive properties of hydrogels

**Fig. 4:**
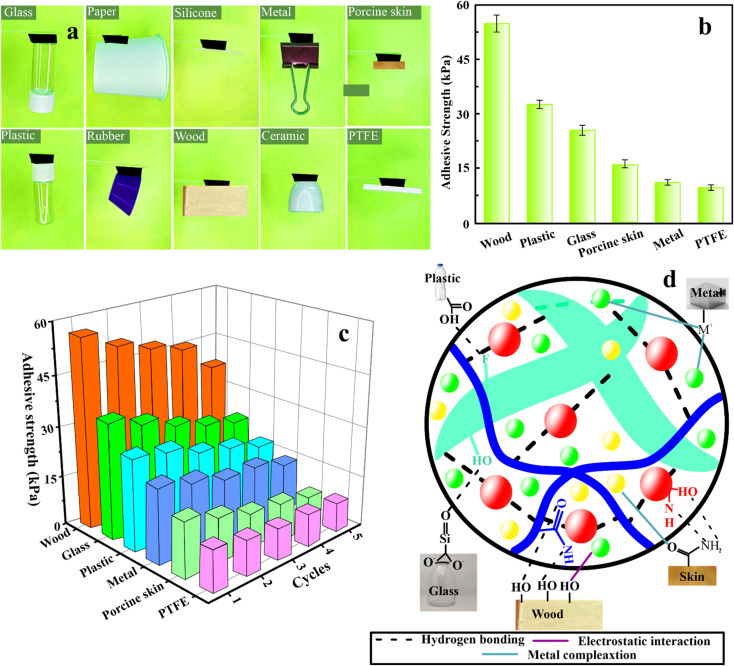
Pictures of the adhesion capability of PAM/MXene_15_/AFP_30_/KCl_15_ to various substrates (a), adhesion strength of the PAM/MXene_15_/AFP_30_/KCl_15_ hydrogel on various substances (b), consecutive adherence–separation cycles on various substrates (c), and possible mechanism of interactions between the PAM/MXene_15_/AFP_30_/KCl_15_ hydrogel and different substrates (d).

PAM/MXene_15_/AFP_30_/KCl_15_ hydrogels displayed outstanding adhesion properties to different hydrophobic and hydrophilic surfaces. Probe-pull tests and lap-shear, which use wood as a substrate, are two mechanical testing approaches that were employed to quantitatively assess the adhesive strength of the hydrogels for wearable strain sensor applications.^29^ Fig. S7(a)† depicts the schematic illustration for the testing procedure. The interfacial shear strength between the substrate and hydrogel was investigated by employing the lap-shear test. Under normal stress conditions, the adhesive strength was assessed by employing the probe-pull test. The outcomes of the lap-shear test for PAM/MXene_15_/AFP_30_/KCl_15_ indicated that the adhesion strength was the highest, achieving 55.3 kPa (Fig. S7(b)).† PAM/MXene_15_/AFP_30_ hydrogel exhibited the second most increased adhesion strength (46.2 kPa), followed by PAM/MXene_15_ (63.5 kPa) and PAM (16.7 kPa). Fig. 4(a) demonstrates that PAM/MXene_15_/AFP_30_/KCl_15_ hydrogel has the ability to efficiently adhere to various substrates, including glass, paper, silicone, metal, porcine skin, plastic, rubber, wood, ceramic, and PTFE. Fig. 4(b) indicates that the maximum adhesion intensities of PAM/MXene_15_/AFP_30_/KCl_15_ hydrogel toward wood, glass, plastic, metal, porcine skin, and PTFE were 55.3, 33.6, 24.5, 15.1, 9.9, and 8.8 kPa, respectively. PAM/MXene_15_/AFP_30_/KCl_15_ hydrogel indicated greater adhesive strength toward wood compared to other substrates, mainly due to the porous and rough surface of wood, which offers greater surface contact area to interact with surface area. The hydrophilic nature of the surface of wood makes it efficiently wettable using the hydrogel, promoting a strong bond *via* hydrogen bonding.^30^ Cyclic lap shear tests were performed on the wood, glass, plastic, metal, porcine skin, and PTFE to examine the reusability of the PAM/MXene_15_/AFP_30_/KCl_15_ hydrogel. Fig. 4(c) displays that the adhesion strength of the hydrogel toward wood, glass, plastic, metal, porcine skin, and PTFE dropped to 80.0%, 78.9%, 82.5%, 96.3%, 70.2%, 63.0%, and 84.5% of the initial value, respectively, after five cycles of successive peeling-adhesion, suggesting that the hydrogel exhibited excellent adhesion reusability. The strong and stabilizing adhesion impact of PAM/MXene_15_/AFP_30_/KCl_15_ hydrogels toward various substrates can be described by the synergistic impact between the hydroxyl, carboxyl, amino, fluoride, and anionic species within the polymer chains. FTIR spectra show PAM/MXene_15_/AFP_30_/KCl_15_ hydrogel consisting of OH, NH, C=O, and CF functional groups (Fig. S1†). These functional groups of hydrogels form different types of bonds with various substrates, including hydrogen bonds, electrostatic interaction, and metal complexation, which enhance the adhesive property of hydrogel (Fig. 4(d)). For example, the abundance of hydroxyl groups in wood allows it to form numerous hydrogen bonds with the hydrogel's hydroxyl and fluoride groups, while the negatively charged oxygen in the hydrogel's hydroxyl groups interacts electrostatically with potassium ions in the hydrogel. Similarly, hydroxyl, amino, and amide groups on the porcine skin surface formed electrostatic interaction and hydrogen bonds with the K^+^, OH, F, and NH groups of the hydrogel. The adhesive forces are strengthened by hydrogen bonds that form between the Si=O groups on the glass surface and the OH groups of hydrogels. Similarly, adhesive forces are strengthened by forming hydrogen bonds, electrostatic interactions, and metal coordination bonds of OH, and metal ions (M^+^) of plastic and metal with OH, NH, F, and K^+^ functional groups of hydrogels.

### 3.5. Conductivity and sensing performance

**Fig. 5:**
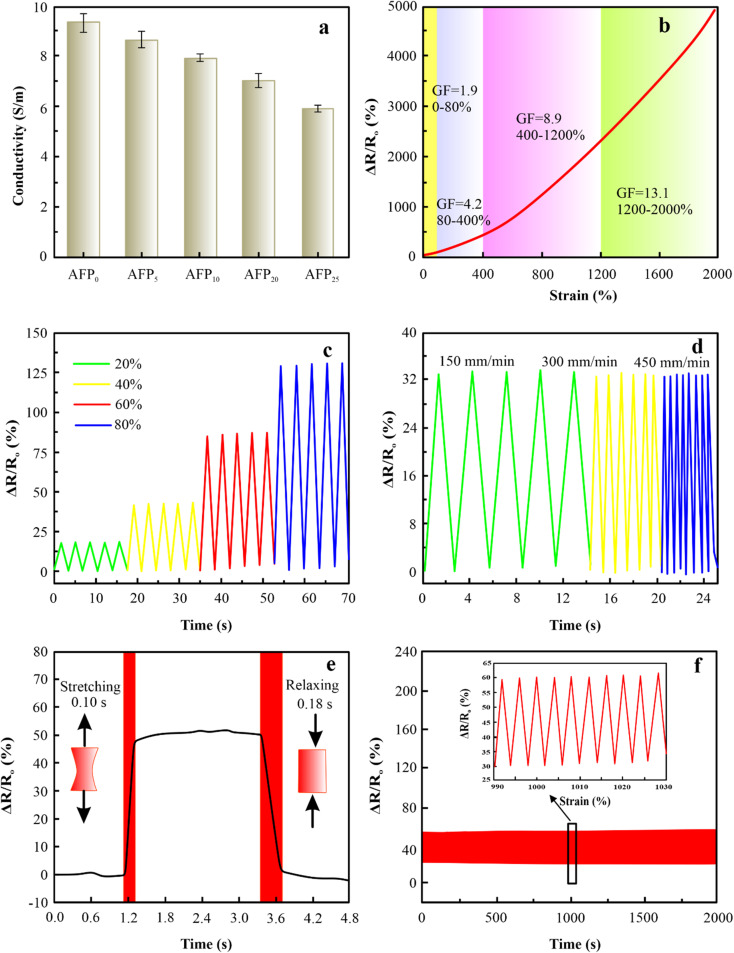
Effect of different concentrations of AFPs on the conductivity (a), sensing performances of the PAM/MXene_15_/AFP_30_/KCl_15_ hydrogel (b), relative resistance changes of the hydrogel-based sensors under various ranges of strain (c), Δ*R*/*R*_0_ of the sensor at various strain levels, (d), response and recovery time of the hydrogel-based sensor (e), and sensing stability of the PAM/MXene_15_/AFP_30_/KCl_15_ hydrogel sensor (f).

The PAM/MXene_15_/AFP_30_/KCl_15_ hydrogel exhibited superior ionic conductivity due to the mobile potassium ions of KCl, which are well suited to designing hydrogel-based strain sensors. The description of the conductivity of hydrogels under variable MXene, AFPs, and KCl concentrations is displayed in Fig. 5(a), S8(a and b).† The rise of MXene, AFPs, and KCl contents led to a drop in ionic conductivity because of the restricted motion caused by free ions due to the dense network of hydrogel.^31^ Despite this, the PAM/MXene_15_/AFP_30_/KCl_15_ hydrogel still exhibited outstanding ionic conductivity, making it a suitable hydrogel-based sensor. The gauge factor (GF) is employed to assess the sensitivity of the hydrogel-based sensor and determined by GF = (Δ*R*/*R*_0_)/*ε*, where Δ*R* denotes resistance in change caused by strain, *R*_0_ represents the original resistance before strain, and *ε* shows applied strain.^32^Fig. 5(b) indicates that the GF values were 1.9 and 4.2 within the strain range of 0–80% and 80–400%, and then significantly growing to 8.9 and 13.1 in a strain range of 400–1200% and 1200–2000%, indicating the high sensitivity of PAM/MXene_15_/AFP_30_/KCl_15_ hydrogel. The excellent responsiveness to strain was attributed to a change in relative resistance caused by the alteration in the structure of the hydrogel. When PAM/MXene_15_/AFP_30_/KCl_15_ hydrogel stretched, the movement of K^+^ was hindered by a thinner ionic conductive path, resulting in an increase in resistance.^33^Fig. 5(c) displays that PAM/MXene_15_/AFP_30_/KCl_15_ hydrogel-based sensors demonstrated effective monitoring of different magnitudes of strains. Additionally, the response-capability of hydrogel-based strain sensors *vs.* stretching rate was studied in Fig. 5(d). As the stretching rate grew, the curves became more denser without a change in relative resistance value. Furthermore, the hydrogel-based sensor indicated response and recovery times of 0.10 s and 0.18 s, respectively, in the stretching and release phases (Fig. 5(d)). As displayed in Fig. 5(f), the hydrogel-based sensor indicated a consistent and stable electrical signal response without evident variations, demonstrating outstanding response stability, which was due to the multiple cross-links of PAM, MXene, AFP, and KCl in the hydrogel network, assuring the reliability and repeatability of the sensing signal.

### 3.6. Anti-icing property

**Fig. 6:**
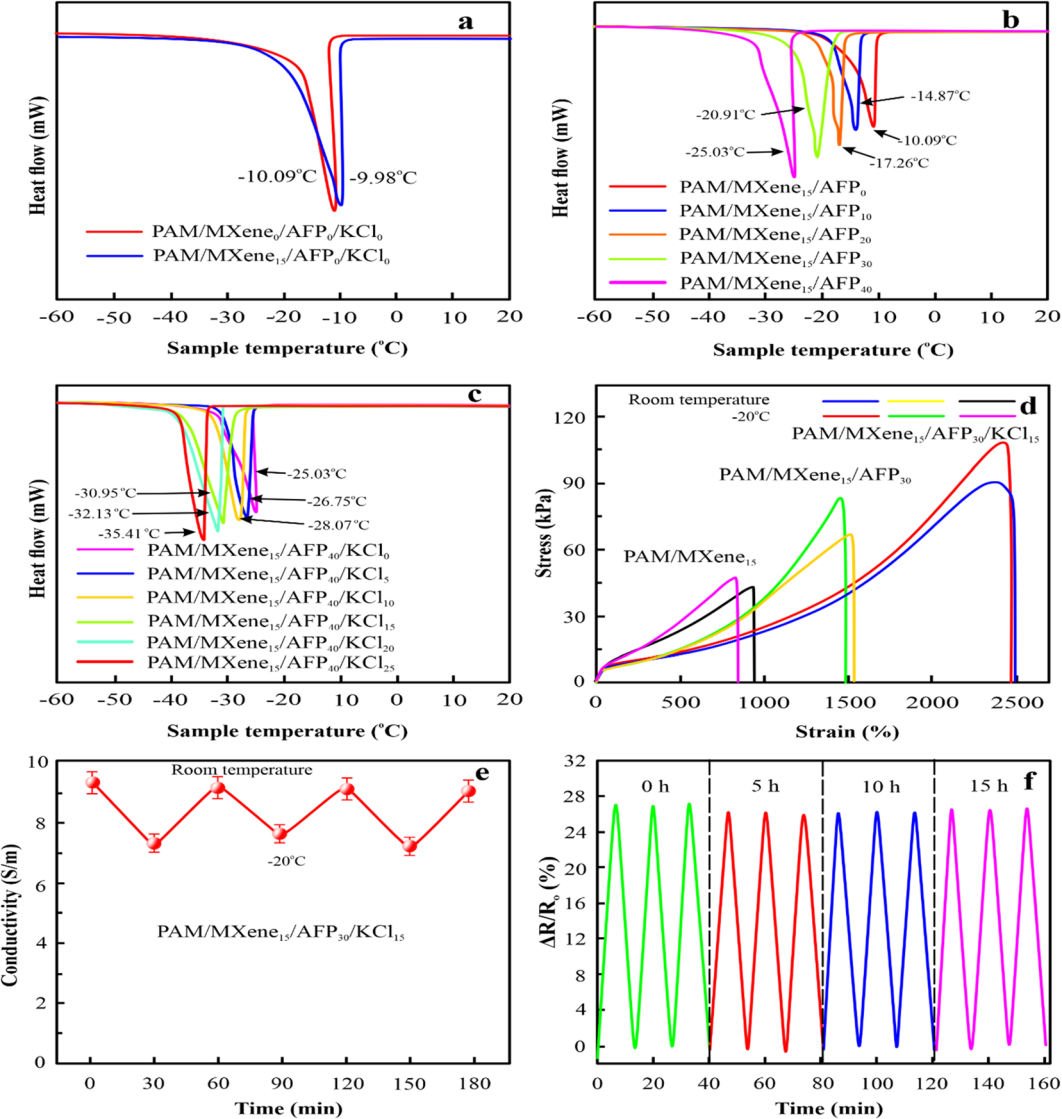
DSC measurements for hydrogels with different MXene contents (a), DSC measurements for PAM/MXene_15_ hydrogels with different AFPs contents (b), DSC measurements for PAM/MXene_15_/AFP_40_ hydrogels with various concentrations of KCl (c), stress and strain curves of the PAM/MXene_15_, PAM/MXene_15_/AFP_30_, and PAM/MXene_15_/AFP_30_/KCl_15_ hydrogels at room temperature and −20 °C (d), conductivity of PAM/MXene_15_/AFP_30_/KCl_15_ hydrogel at room temperature and −20 °C (e), Δ*R*/*R*_0_ of PAM/MXene_15_/AFP_30_/KCl_15_ hydrogel an after placing at −20 °C for 0, 5, 10 and 15 hours (f).

Due to water crystallization, traditional conductive hydrogels tend to harden and freeze at temperatures below the freezing point, which limits their use in cold circumstances. To address this issue, MXene, AFP, and KCl were added to the hydrogels to make hydrogels with remarkable anti-icing properties in low-temperature conditions. The as-synthesized hydrogels were kept at −20 °C for 24 h. DSC investigations were performed to study the anti-icing capability of prepared hydrogels. Fig. 6(a) displays that the icing point of the PAM/MXene_0_ and PAM/MXene_15_ hydrogel were −9.98 °C and −10.09 °C, respectively. With the introduction of 0, 10, 20, 30, and 40 wt% AFPs in the PAM/MXene_15_ hydrogel, the icing temperature of the related hydrogel was −10.09, −14.87, −17.26, −20.91, and −25.03 °C respectively, demonstrating the introduction of AFP could effectively suppress ice crystal formation in the hydrogel (Fig. 6(b)).^34,35^ With the addition of 0, 5, 10, 15, 20, and 25 wt% KCl in the PAM/MXene_15_/AFP_40_ hydrogel, the related hydrogel's freezing temperature was −25.03, −26.75, −28.07, −30.95, −32.13, and −35.41 °C, respectively, demonstrating the introduction of KCl results in the decrease of the freezing temperature, which caused by the suppressing of ice crystals formation by the KCl (Fig. 6(c)).^36^ The outcome indicated that the increase in concentrations of AFP and KCl efficiently decreased the freezing temperature of the hydrogel. As depicted in Fig. 6(d), the tensile stress and strain curves of PAM/MXene_15_, PAM/MXene_15_/AFP_30_, and PAM/MXene_15_/AFP_30_/KCl_15_ hydrogels. Fig. 6(d) indicates that there is practically no variation in the mechanical strength between −20 °C and room temperature for hydrogel. Although the ionic conductivity of hydrogels decreased in low-temperature environments, they still retained remarkable electrical properties and exhibited stable cycle performance at different temperatures. As displayed in Fig. 6(e). Moreover, the sensing capability of PAM/MXene_15_/AFP_30_/KCl_15_ hydrogel was carried out after storing it at −20 °C for different time intervals (Fig. 6(f)). Evidently, there is no notable change in the relative resistance of PAM/MXene_15_/AFP_30_/KCl_15_ hydrogel.

### 3.7. Strain-sensing performances of PAM/MXene_15_/AFP_30_/KCl_15_ hydrogels

**Fig. 7:**
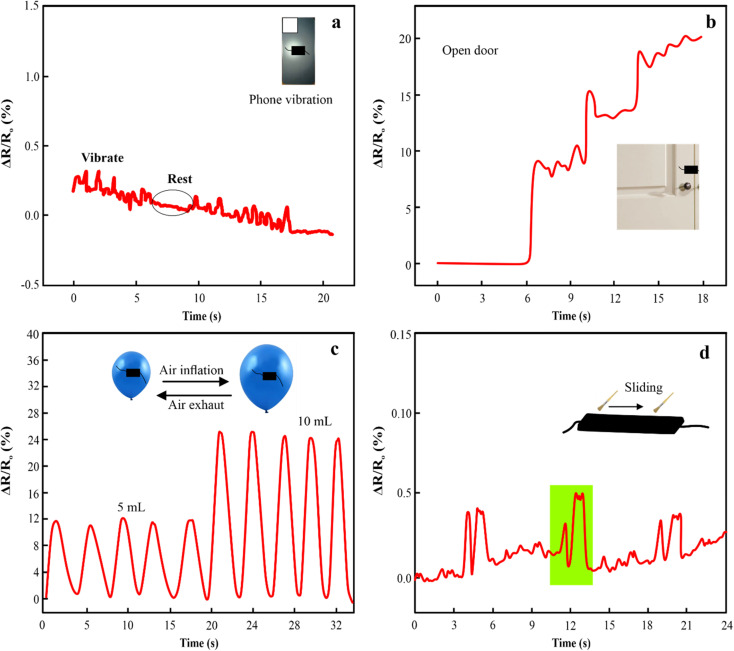
Δ*R*/*R*_0_ of PAM/MXene_15_/AFP_30_/KCl_15_ hydrogel during rest and vibration of mobile phone (a), relative resistance changes of PAM/MXene_15_/AFP_30_/KCl_15_ hydrogel caused by the opening of a wooden door (b), relative resistance changes of PAM/MXene_15_/AFP_30_/KCl_15_ hydrogel sensor caused by injecting a distinct volume of air into the plastic balloon (c), Δ*R*/*R*_0_ due to sliding brush on the PAM/MXene_15_/AFP_30_/KCl_15_ hydrogel's surface (d).

The PAM/MXene_15_/AFP_30_/KCl_15_ hydrogels exhibited outstanding mechanical properties, adhesive strength, ionic conductivity, and anti-icing properties, which make them well-suited for preparing strain sensors. The relative resistance (RS) of PAM/MXene_15_/AFP_30_/KCl_15_ hydrogel changes as the mobile phone vibrates. The PAM/MXene_15_/AFP_30_/KCl_15_ hydrogel indicated a sharp change in the relative resistance during vibrations of mobile phones and exhibited flat changes when the phone was not vibrated (Fig. 7(a)). Fig. 7(b) displays the PAM/MXene_15_/AFP_30_/KCl_15_ hydrogel sensors placed on both sides of the wooden door. Upon opening the door, there was a change in PAM/MXene_15_/AFP_30_/KCl_15_ hydrogel sensor length, which increased relative resistance. Similarly, the PAM/MXene_15_/AFP_30_/KCl_15_ hydrogel sensor was placed on the ballon's surface to observe inflation by injecting distinct 5 mL and 10 mL volumes. The elongation of hydrogel generated by the expansion of the balloon resulted in notable growth in the relative resistance changes (Fig. 7(c)). The PAM/MXene_15_/AFP_30_/KCl_15_ hydrogel sensors have the ability to repeatedly and precisely monitor the changes in air pressure, confirming the practical uses in nonplanar-based strain sensing. The relative resistance improved when the brush slid on the surface of the PAM/MXene_15_/AFP_30_/KCl_15_ sensor and then dropped as the brush stopped sliding (Fig. 7(d)).

**Fig. 8:**
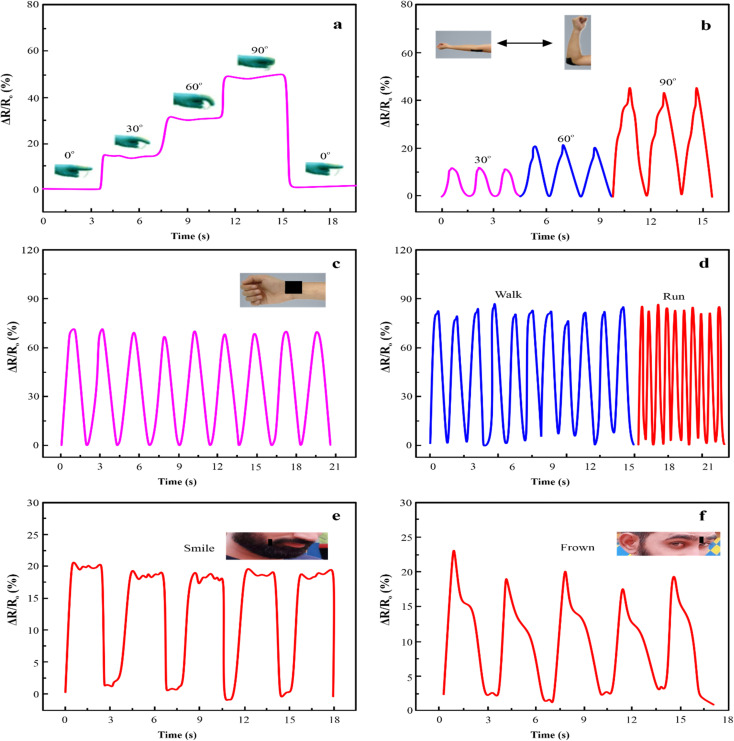
Relative resistance changes of PAM/MXene_15_/AFP_30_/KCl_15_ hydrogel-based strain sensor for detection of finger bending at distinct angles (a), elbow flexing at distinct angles (b), and wrist bending (c). Relative resistance changes of PAM/MXene_15_/AFP_30_/KCl_15_ hydrogel-based strain sensor during running and walking. Relative resistance changes of PAM/MXene_15_/AFP_30_/KCl_15_ hydrogel-based strain sensor during smiling (e) and frowning (f).

According to the numerous benefits of the PAM/MXene_15_/AFP_30_/KCl_15_ hydrogel strain sensor, it can be utilized as a wearable sensor to observe the human body's movements. The PAM/MXene_15_/AFP_30_/KCl_15_ hydrogel sensor was attached to the knuckle and determined finger flexion at different angles of 0°, 30°, 60°, and 90° by observing the change in resistance (Fig. 8(a)). It is important that, upon release of stress, the resistance rapidly falls back to its original value. Furthermore, the PAM/MXene_15_/AFP_30_/KCl_15_ hydrogel strain sensor has the ability to identify the elbow and wrist motions of the volunteer (Fig. 8(b and c)). When recording the elbow movements, the resistance was enhanced with a rising bending angle, suggesting that the PAM/MXene_15_/AFP_30_/KCl_15_ hydrogel sensor has the ability to identify various ranges of movement of the same joint cycles (Fig. 8(b)). When recording wrist flexion, the strain PAM/MXene_15_/AFP_30_/KCl_15_ hydrogel sensor shows a reproducible and significant response under numerous flexion-release cycles (Fig. 8(c)). This indicates the excellent stability of the PAM/MXene_15_/AFP_30_/KCl_15_ hydrogel sensor. Furthermore, the ternary hydrogel sensor was attached to the knee joint to record intense movement, such as running and walking. With the increase in movement speed, the signals became accordingly denser, and the hydrogel sensor exhibited good reliability and repeatability (Fig. 8(d)). In addition to the detection of human joint movements, the PAM/MXene_15_/AFP_30_/KCl_15_ hydrogel has the ability to detect the muscle's movements. Thus, the hydrogel sensors were immobilized on the mouth corners and foreheads of the volunteers to detect muscle motions close to the skin. As illustrated in Fig. 8(d and e), the PAM/MXene_15_/AFP_30_/KCl_15_ hydrogel-based sensor generates distinctive resistance responses to smiling and frowning, which suggests that the sensor can be used as an emotion monitor for recording emotional expression.

Distinct tactile perception plays an essential role in the advanced applications of the hydrogel-based sensor. The PAM/MXene_15_/AFP_30_/KCl_15_ hydrogel was fastened to the thumb, acting as a tactile sensor. The tactile sensor was utilized to differentiate the object's hardness in Fig. S9(a).† The output response signals displayed a greater amplitude when the hand touched a harder iron ball because of the tighter contact force compared to a soft yarn ball. Such a result displayed that the PAM/MXene_15_/AFP_30_/KCl_15_ hydrogel sensor indicated sufficient sensitivity to distinguish the weight of bodies. The amplitude of the curve of the hydrogel sensor was significantly higher when holding a 50 g weight compared to the 25 g (Fig. S9(b)†). Moreover, Fig. S10† exhibits that the PAM/MXene_15_/AFP_30_/KCl_15_ hydrogel was affixed to the human skin for five days, and upon removal, no skin inflammation or damage was observed, highlighting its exceptional skin compatibility. Reproducible and stable signals were achieved when the hand repeatedly grasped and touched objects, suggesting the trustworthy and promising capability of the PAM/MXene_15_/AFP_30_/KCl_15_ hydrogel for use in soft robots as artificial skin.

The feasibility of PAM/MXene_15_/AFP_30_/KCl_15_ hydrogel sensors for observing unexpected movements in animals, employing the mouse as a sample. For the preparation of wearable sensors, metal wires hydrogel integrated with PAM/MXene_15_/AFP_30_/KCl_15_ hydrogel. The PAM/MXene_15_/AFP_30_/KCl_15_ hydrogel was attached to the mouse's body, and the metal wires protruding from the PAM/MXene_15_/AFP_30_/KCl_15_ were integrated with the workstation. As illustrated in Fig. S11(a),† the PAM/MXene_15_/AFP_30_/KCl_15_ hydrogel was attached to the mouse's tail to detect the wagging of the tail. A minor signal variation was recorded as the mouse swung its tail gently. Furthermore, when the mouse was grabbed unexpectedly, abrupt shock encouraged the mouse to wildly wag its tail, leading to notable growth in relative resistance changes. Respiration patterns in various conditions were also recorded by PAM/MXene_15_/AFP_30_/KCl_15_ hydrogel-based sensors positioned on the mouse's chest. Notable and reproducible output signals of resistance changes were generated during the mouse's inhalation and exhalation process, as exhibited in Fig. S11(b).† Fig. S11(c)† displays that the relative resistance of hydrogel changed as the mouse ran and primarily held steady as the mouse was in a rest state. Changes in the relative resistance were caused by the elongation of the PAM/MXene_15_/AFP_30_/KCl_15_ hydrogel due to the motion of the mouse's leg. It is essential to note that the PAM/MXene_15_/AFP_30_/KCl_15_ hydrogel sensor had the ability to detect the physiological responses of animals in various conditions, thereby assisting in understanding the fundamental biological mechanisms of specific behavioral phenomena.^37^

## 4 Conclusion

In summary, we prepared a tunable hydrogel characterized by freeze-tolerance properties by incorporating MXene, AFPs, and KCl into the PAM polymer network. The resulting hydrogels exhibited excellent self-adhesion, robust mechanical strength, and ultra-stretchability. As expected, the introduction of KCl and AFPs endowed a hydrogel with anti-icing properties. The resulting PAM/MXene_15_/AFP_30_/KCl_15_ hydrogel demonstrated negligible hysteresis behavior, quick electromechanical response (0.10 s), and excellent sensitivity (GF = 13.1 within the strain range of 1200–2000%). Thus, PAM/MXene_15_/AFP_30_/KCl_15_ hydrogels could serve as sensors for accurately detecting animal or human movements and physiological motions, including different joint bending, larynx vibration, and respiratory signals. The approach of developing materials with tunable features, along with inorganic salt and the fish-inspired freeze-tolerance method, offers a new prospect for wearable gadgets.

## Ethical statement

The Code of Ethics of the World Medical Association (Declaration of Helsinki) for experiments involving humans, and in line with the Recommendations for the Conduct, Reporting, Editing and Publication of Scholarly Work in Medical Journals. The informed consent was obtained for experimentation with human subjects. The privacy rights of human subjects were observed.

## Data availability

Data will be made available on request.

## Author contributions

Aysha Bukhari: conceptualization, visualization, supervision, writing original draft, reviewing and editing, Irfan Ijaz: conceptualization, methodology, investigation, writing original draft, and supervision, Ezaz Gilani: resource, software, and methodology, Ammara nazir: data curation, formal analysis, and investigation, Hina Zain: data curation and investigation, Attia Shaheen: methodology, and data curation, Mohammed Rafi Shaik; data curation, formal analysis, Mohamed E. Assal; data curation, methodology, Mujeeb Khan; funding acquisition, investigation, visualization.

## Conflicts of interest

The authors declare that they have no known competing financial interests or personal relationships that could have appeared to influence the work reported in this paper.

## Supplementary Material

RA-014-D4RA02707H-s001
