# Supplementary material for: MXene, protein, and KCl-assisted ionic conductive hydrogels with excellent anti-freezing capabilities, self-adhesive, ultra-stretchability, and remarkable mechanical properties for a high-performance wearable flexible sensor
Source: RSC Adv. 2024 Jul 9;14(30):21786–98. doi: 10.1039/d4ra02707h (PMC11231829; doi:10.1039/d4ra02707h)
Supplement: RA-014-D4RA02707H-s001 [file RA-014-D4RA02707H-s001.pdf]

Electronic Supplementary Material (ESI) for RSC Advances.
This journal is © The Royal Society of Chemistry 2024

Supplementary Material

MXene, Protein, and KCl-assisted ionic conductive hydrogels with excellent anti-freezing capabilities, self-adhesive, ultra-stretchability, and remarkable mechanical properties for high-performance wearable flexible sensor

Irfan Ijaz[1*], Aysha Bukhari[1*], Ezaz Gilani[1], Ammara Nazir[1], Dr. Hina Zain[2], Attia Shaheen[3], Mohammed Rafi Shaik[4], Mohamed E. Assal[4], Mujeeb Khan[4],

[1]School of Chemistry, Faculty of Basic Sciences and Mathematics, Minhaj University Lahore, Lahore 54700, Pakistan

[2] Department of chemistry, University of Cincinnati, OH 45221, United States

[3]Institute for Advanced Study, Shenzhen University, Shenzhen, Guangdong, P.R. China

[4]Department of Chemistry, College of Science, King Saud University, P.O. Box 2455, Riyadh 11451, Saudi Arabia

Corresponding Authors;

Irfan Ijaz; iffichemixt266@gmail.com

Aysha Bukhari: ayshabukhari.che@mul.edu.pk

Text S1. Materials

MAX powder (Ti_3AlC_2) and antifreeze proteins (AFPs) were purchased from Waltham, MA; USA. Ammonium persulfate (APS), N, N′ methylenebisacrylamide (MBAA), and potassium chloride (KCl) were obtained from Shanghai Aladdin Co., Ltd.

Text S2. Preparation of MXene suspension

MXene was synthesized through etching MAX powder (Ti_3AlC_2) with LiF and HCl. Particularly, 2 g of LiF was mixed with 20 mL of 10M HCl solution and stirred for 10 min in a Teflon beaker. Then, 3 g of Ti_3AlC_2 MAX powder was gradually introduced into the mixed solution and reacted at 45 °C for 20 h. The mixture was then centrifuged at 3500 rpm for 10 minutes and rinsed multiple

times to neutrality with DI water. The obtained mixture was centrifuged with deionized water at 4500 rpm for 10 min, and the resulting supernatant was poured off. This phenomenon was repeated until the color of the supernatant turned black. MXene sediments were collected by applying a greater centrifugal speed of 7500 rpm. To prepare the MXene suspension, the previously collected sediments were introduced into 150 mL of deionized water and then sonicated in an Ar environment for 60 min, finally again centrifuged for 60 min at 3500 rpm.

Text S3. Adhesion test

The adhesive strengths of hydrogel with different substrates were determined using lap shear tests by employing the CT3-1000 texture analyzer (USA). The PAM/MXene/AFPs/KCl hydrogels were cut into 10 mm × 10 mm and placed between two identical substrates affixed to the plates. The adhesive strength of hydrogel was calculated by splitting the maximum load by the initial area of adhesion[1]. Glass, paper, silicone, metal, porcine skin, plastic, rubber, wood, ceramic, and PTFE were chosen as substrates to explore the universal adhesion property of hydrogels.

Text S4. Conductivity analysis

The electrochemical workstation (TH2831, made in China) was utilized to describe the conductivity of the hydrogel, which comprised differing concentrations of MXene, AFPs, and KCl. The conductivity of hydrogel was calculated by the following formula:

$$\sigma = L / R \times S$$ * MERGEFORMAT (1)

Where S (cm^2), R (Ω), and L (cm) denote the cross-sectional area, resistance, and test length of hydrogel, respectively.

Text S5. Mechanical test

The mechanical strength of PAM/MXene/AFPs/KCl hydrogels was examined by a multifunctional testing machine with a 100 N load sensor (Shimadzu AGS-X, Japan). The PAM/MXene/AFPs/KCl hydrogels were prepared in dumbbell-like shape with thickness of 3 mm and width of 4 mm.

Text S6. DSC analysis

A differential scanning calorimeter (DSC25, TA, made in the USA) was used to investigate the freeze resistance of PAM/MXene/AFPs/KCl hydrogel at low temperatures. The DSC analysis started at a temperature of 20 °C and then decreased to -60 °C.

Text S7. Electromechanical measurements

The electrochemical workstation (Vertex C, IVIUM Tech, made in the Netherlands) connected to the tensile tester (MCT-2150, A&D, made in Japan) was employed to investigate the electromechanical response of the hydrogels. The electrochemical workstation recorded output signals as hydrogel was stretched by using a tensile tester. However, the PAM/MXene/AFPs/KCl hydrogels were immobilized on the human body to serve as hydrogel-based sensors to monitor bodily motions by attaching them to metal wires. The equation given below was employed to record the output signal.

$$\Delta R / R_0 (\%) = (R - R_0) \times 100 \qquad \text{* MERGEFORMAT (2)}$$

where R_0 and R denote resistance before and after applying strain on the PAM/MXene/AFPs/KCl hydrogel.

Table S1: Recipes for the preparation of all hydrogel specimens

**Hydrogels**	**AM (g)**	**MBAA (g)**	**APS (g)**	**MXene (g)**	**AFP (g)**	**KCl (g)**	**Water (mL)**
PAM/MXene$_{0}$	2	0.0010	0.05	0	-	-	20
PAM/MXene$_{5}$	2	0.0010	0.05	0.084	-	-	20
PAM/MXene$_{10}$	2	0.0010	0.05	0.168	-	-	20
PAM/MXene$_{15}$	2	0.0010	0.05	0.251	-	-	20
PAM/MXene$_{20}$	2	0.0010	0.05	0.335	-	-	20
PAM/MXene$_{20}$/AFP$_{0}$	2	0.0010	0.05	0.251	0	-	20
PAM/MXene$_{20}$/AFP$_{10}$	2	0.0010	0.05	0.251	2.3	-	20
PAM/MXene$_{20}$/AFP$_{20}$	2	0.0010	0.05	0.251	4.6	-	20
PAM/MXene$_{20}$/AFP$_{30}$	2			0.251	6.9	-	20
PAM/MXene$_{20}$/AFP$_{40}$	2	0.0010	0.05	0.251	9.2	-	20
PAM/MXene$_{20}$/AFP$_{30}$/KCl$_{0}$	2	0.0010	0.05	0.251	6.9	-	20
PAM/MXene$_{20}$/AFP$_{30}$/KCl$_{5}$	2	0.0010	0.05	0.251	6.9	0.0373	20
PAM/MXene$_{20}$/AFP$_{30}$/KCl$_{10}$	2	0.0010	0.05	0.251	6.9	0.075	20
PAM/MXene$_{20}$/AFP$_{30}$/KCl$_{15}$	2	0.0010	0.05	0.251	6.9	0.112	20
PAM/MXene$_{20}$/AFP$_{30}$/KCl$_{20}$	2	0.0010	0.05	0.251	6.9	0.149	20
PAM/MXene$_{20}$/AFP$_{30}$/KCl$_{25}$	2	0.0010	0.05	0.251	6.9	0.186	20

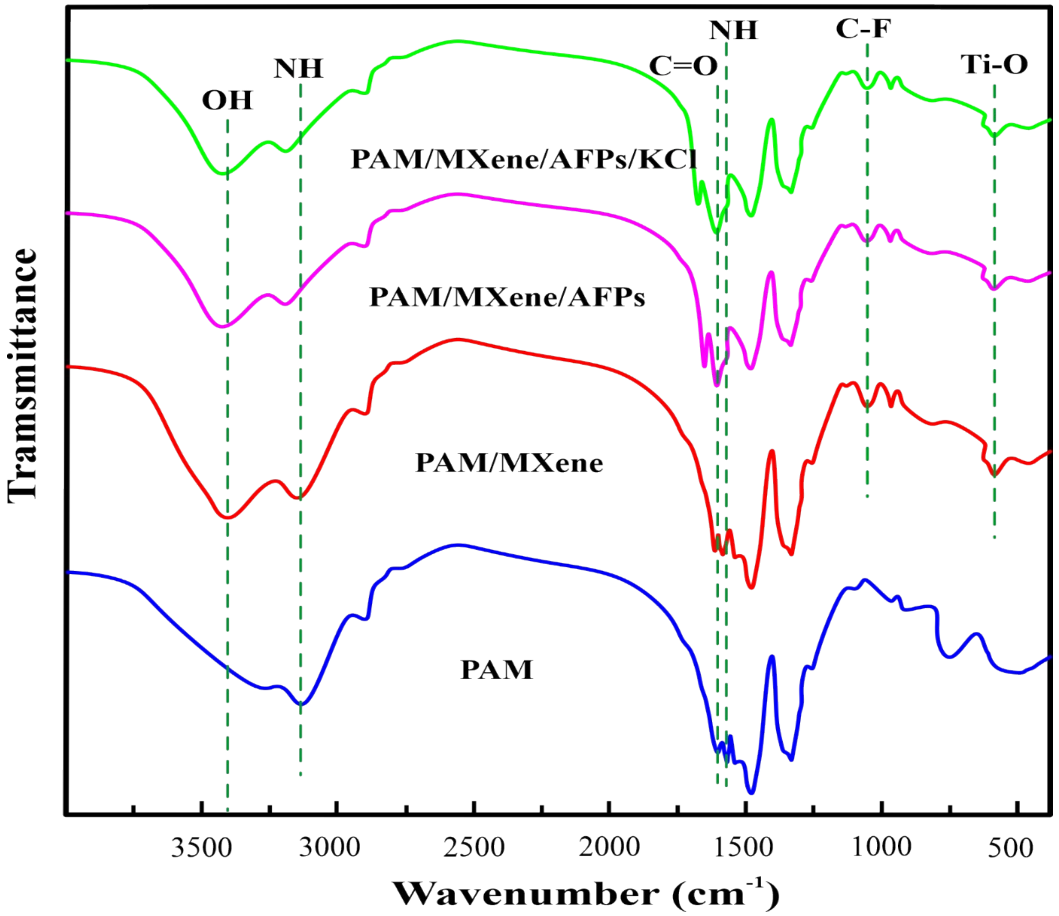

Figure S1: FTIR spectra of pristine PAM, PAM/MXene, PAM/MXene/APFs, and PAM/MXene/APFs/KCl hydrogels

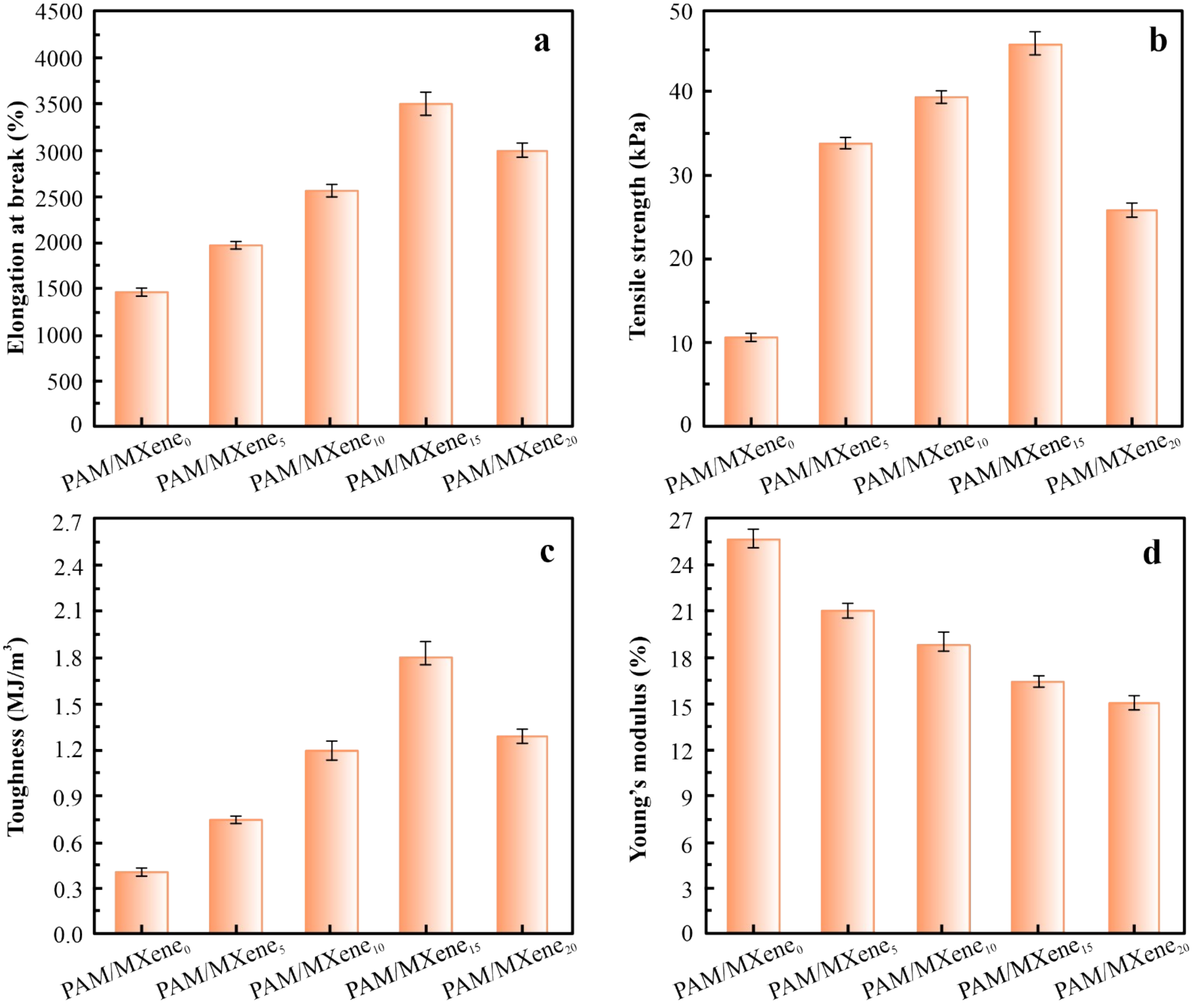

Figure S2: Elongation at break (a), tensile strength (b), toughness (c), and Young's modulus of hydrogels with varying MXene contents

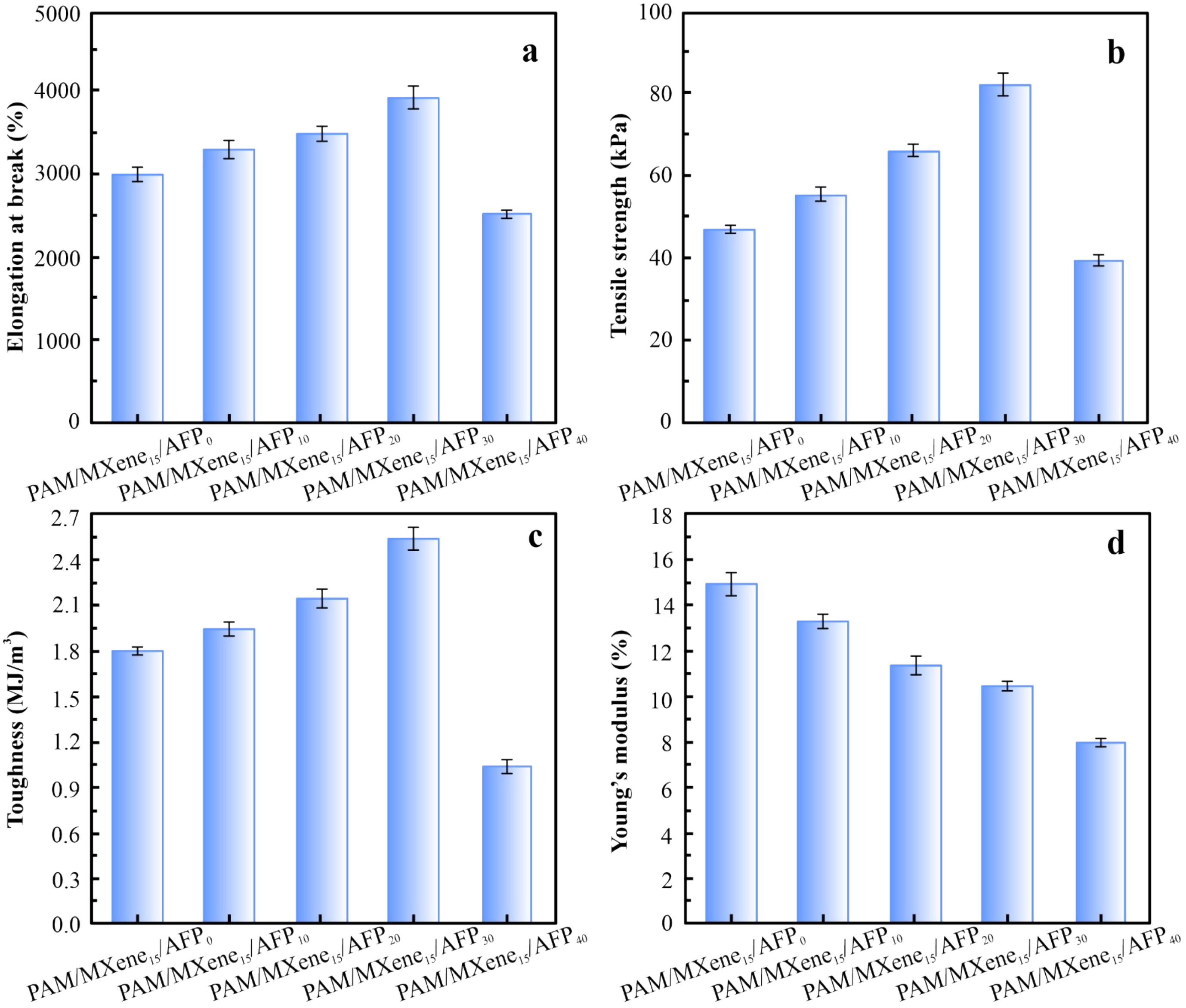

Figure S3: **Elongation at break (a), tensile strength (b), toughness (c), and Young's modulus of hydrogels with varying AFPs contents**

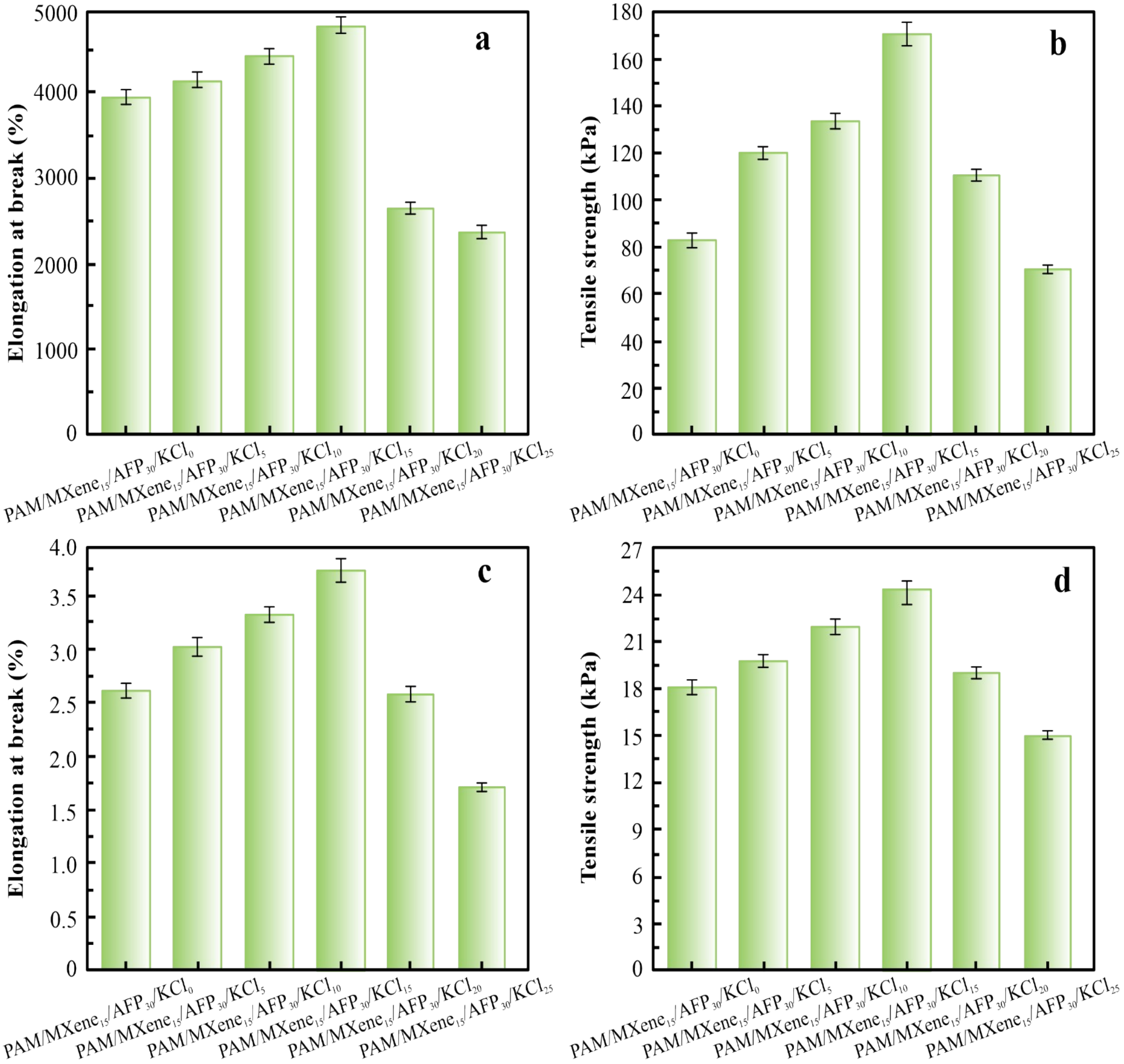

Figure S4: Elongation at break (a), tensile strength (b), toughness (c), and Young's modulus of hydrogels with varying KCl contents

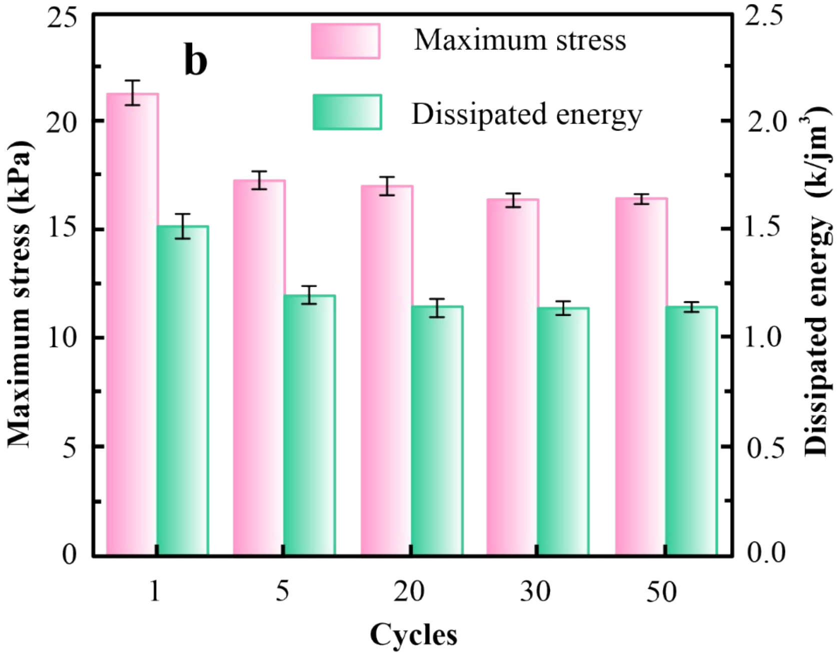

Figure S5: Maximum stress and dissipation energy in cyclic tensile curves of PAM/MXene$_{15}$/AFP$_{30}$/KCl$_{15}$ hydrogel for 50 cycles

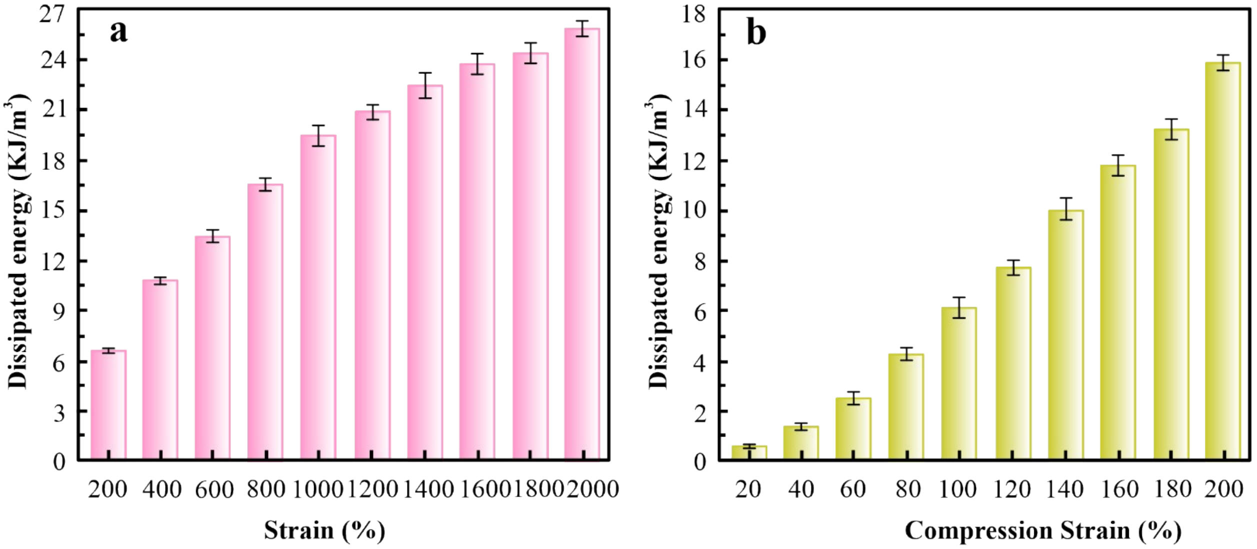

Figure S6: **Dissipated energy at various strains in the continuous cyclic tensile curves of the PAM/MXene$_{15}$/AFP$_{30}$/KCl$_{15}$ hydrogel (a) and dissipated energy at various strains in the continuous cyclic compressive curves of the PAM/MXene$_{15}$/AFP$_{30}$/KCl$_{15}$ hydrogel (b)**

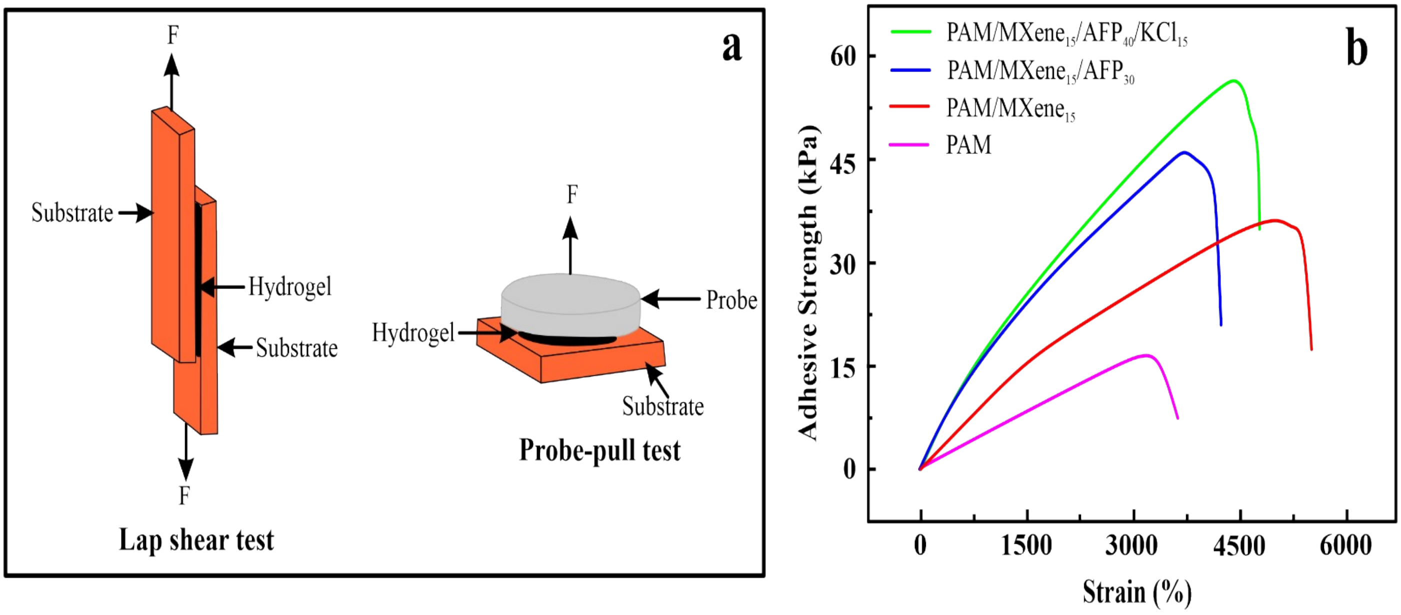

Figure S7: Diagrammatic representation of the experimental configuration for the probe-pull and lap-shear tests (a) and adhesion strengths of PAM, $PAM/MXene_{15}$, $PAM/MXene_{15}/AFP_{30}$, and $PAM/MXene_{15}/AFP_{30}/KCl_{15}$.

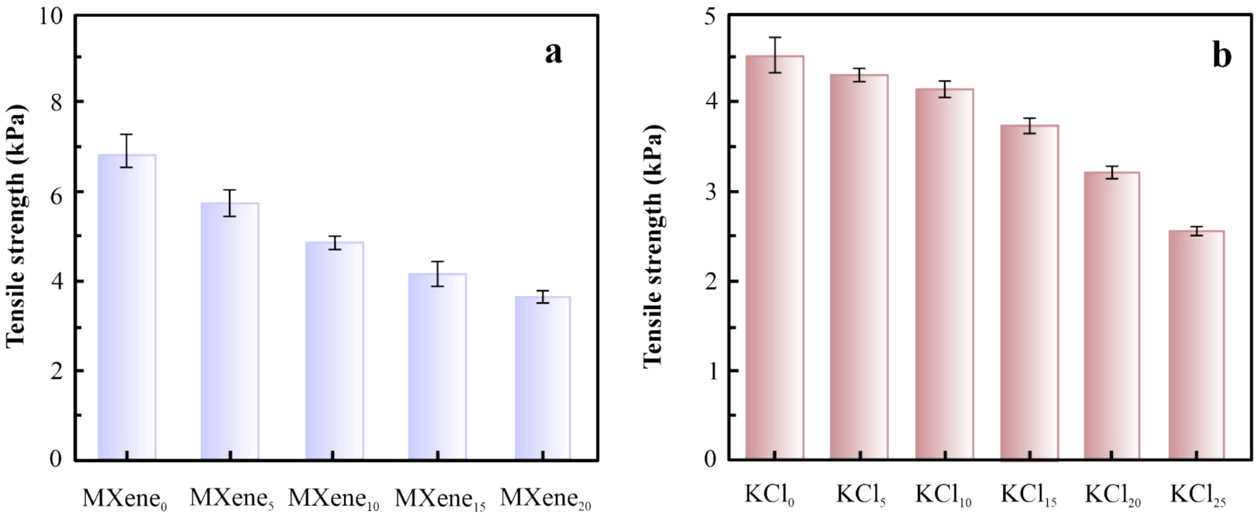

Figure S8: Effect of different MXene contents on the conductivity of hydrogels (a), and Effect of different KCl contents on the conductivity of hydrogels.

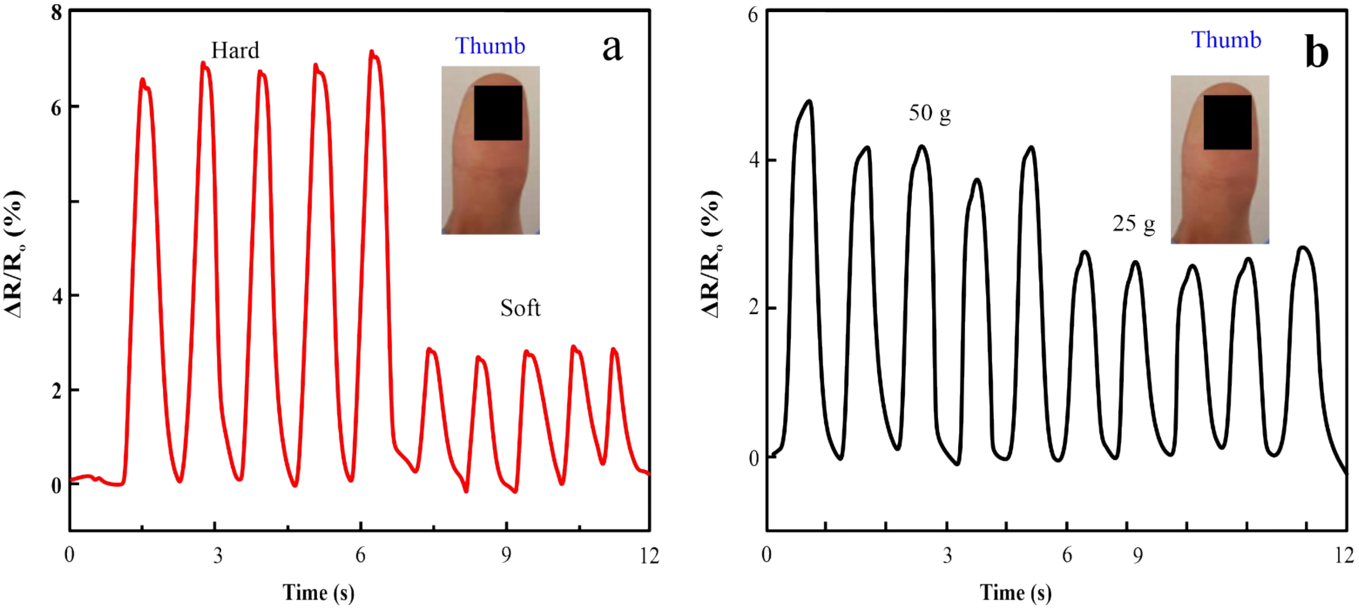

Figure S9: relative resistance changes of hydrogel-based sensors as the volunteer touched the iron hard ball or soft yarn ball (a) and captured various weights (b)

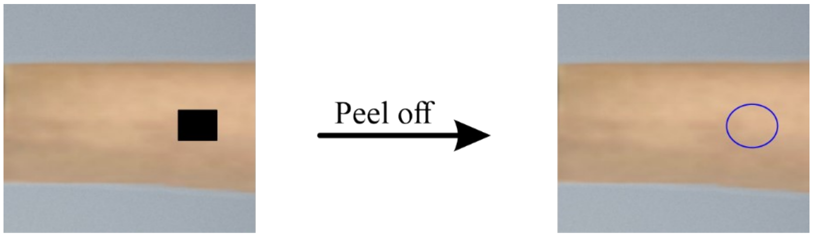

Figure S10: The display of skin affinity ability of PAM/$MXene_{15}$/AFP_{30}/KCl_{15} hydrogel after affixed on human skin for 5 days.

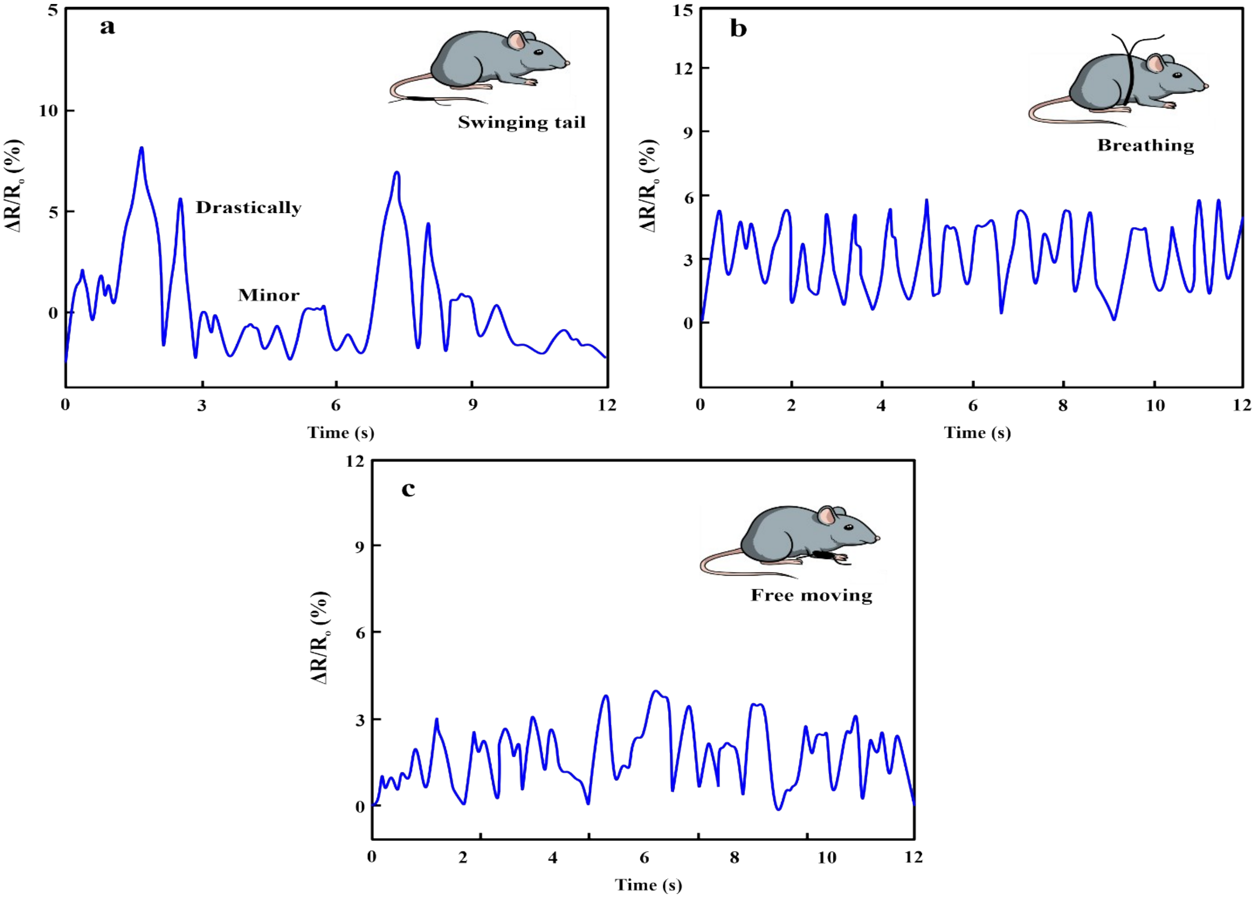

Figure S10: Electrical output signals of PAM/MXene$_{15}$/AFP$_{30}$/KCl$_{15}$ hydrogel-based sensors for detection of tail swinging of mouse (a), ΔR/R$_0$ curves versus time of PAM/MXene$_{15}$/AFP$_{30}$/KCl$_{15}$ hydrogel-based sensor affixed on a mouse leg for monitoring free movement (b), respiratory output signals from the PAM/MXene$_{15}$/AFP$_{30}$/KCl$_{15}$ hydrogel-based sensor under the painful and normal condition (c)

References

1 R. Song, X. Wang, M. Johnson, C. Milne, A. Lesniak-Podsiadlo, Y. Li, J. Lyu, Z. Li, C. Zhao and L. Yang, *Adv. Funct. Mater.*, 2024, 2313322.
